# Upsurge of Enterovirus D68 and Circulation of the New Subclade D3 and Subclade B3 in Beijing, China, 2016

**DOI:** 10.1038/s41598-019-42651-7

**Published:** 2019-04-15

**Authors:** Lingyu Shen, Cheng Gong, Zichun Xiang, Tiegang Zhang, Maozhong Li, Aihua Li, Ming Luo, Fang Huang

**Affiliations:** 1 School of Public Health, Capital Medical University, Beijing, 100069 P.R. China; 2 Institute for immunization and prevention, Beijing Municipal Center for Disease Prevention and Control, Beijing, 100013 P.R. China; 3 MOH Key Laboratory of Systems Biology of Pathogens and Christophe Mérieux Laboratory, IPB, CAMS-Foundation Mérieux, Institute of Pathogen Biology (IPB), Chinese Academy of Medical Sciences (CAMS) & Peking Union Medical College, Beijing, 100730 P.R. China

## Abstract

We conducted a surveillance among acute respiratory tract infection (ARTI) cases to define the epidemiology, clinical characteristics and genetic variations of enterovirus D68 (EV-D68) in Beijing, China from 2015 to 2017. Nasopharyngeal swabs and sputum were collected from 30 sentinel hospitals in Beijing and subjected to EV and EV-D68 detection by real-time PCR. The VP1 gene region and complete genome sequences of EV-D68 positive cases were analyzed. Of 21816 ARTI cases, 619 (2.84%) were EV positive and 42 cases were EV-D68 positive. The detection rates of EV-D68 were 0 (0/6644) in 2015, 0.53% (40/7522) in 2016 and 0.03% (2/7650) in 2017, respectively. Two peaks of EV-D68 infections occurred in late summer and early-winter. Ten cases (23.81%) with upper respiratory tract infection and 32 cases (76.19%) presented with pneumonia, including 3 cases with severe pneumonia. The phylogenetic analysis suggested 15 subclade D3 strains and 27 subclade B3 strains of EV-D68 were circulated in China from 2016 to 2017. A total of 52 amino acid polymorphisms were identified between subclades D1 and D3. These data suggest an upsurge of EV-D68 occurred in Beijing in 2016, the new subclade D3 emerged in 2016 and co-circulated with subclade B3 between 2016 and 2017.

## Introduction

Enteroviruses (EV) are small, non-enveloped viruses with a single-stranded, positive-sense RNA in the family *Picornaviridae*^1,2^. The length of enteroviruses is approximately 7.5 kilobases, and divided into 15 species (EV-A to L and rhinovirus A to C)^3–6^. Seven species, including EV-A to D and rhinovirus (RV) A to C, caused a wide spectrum of diseases in human^7,8^.

Enterovirus D68 (EV-D68), a member of the species EV-D, was first identified from bronchiolitis and pneumonia in California in 1962^9^. EV-D68 infections have been rarely reported until the worldwide outbreaks^10–18^. A nationwide outbreak of EV-D68 associated with severe respiratory tract infection was reported in the USA from mid-August 2014 to January 15, 2015 and 1,153 cases were confirmed with clinical characteristics of patients mainly resembling pneumonia with severe dyspnea syndrome during this epidemic^19^. In China, sporadic cases of EV-D68 were reported between 2006 and 2014^13,20–23^. The similar results were found that few patients confirmed with EV-D68 infections during the surveillance among US^24^, China^21^ in 2015. However, an upsurge of EV-D68 infections occurred in 2016, ranging from Europe to North America, including Netherlands, France, Sweden and United states^24–27^. In addition, sporadic cases have been reported in Hong Kong, Italy, Germany, Portugal and UK in 2016^28,29^.

Earlier phylogenetic analysis revealed that EV-D68 strains circulating worldwide during 1961–2013 were divided into three clades (clades A to C) or lineages (lineages 1 to 3)^10,11,29–31^. With the deepening of the study, the clades of EV-D68 were further refined. After the outbreak of the United States in 2014, EV-D68 was grouped into four clades and five subclades, including clade A, clade B (subclades B1 to B3), clade C and clade D (subclades D1 to D2)^32,33^. The subclade B1 and B3, as the major subclade, circulated around the world in 2014 and in 2016, respectively.

To raise awareness of this upsurge of EV-D68 infection, we performed an EV-D68 surveillance study on ARTI cases from the Respiratory Virus Surveillance System (RVSS) in Beijing and analyzed the epidemiological and clinical characteristics of EV-D68 infections from 2015 to 2017 in Beijing.

## Results

### Detection of EV-D68 infections from 2015 to 2017

**Figure 1:**
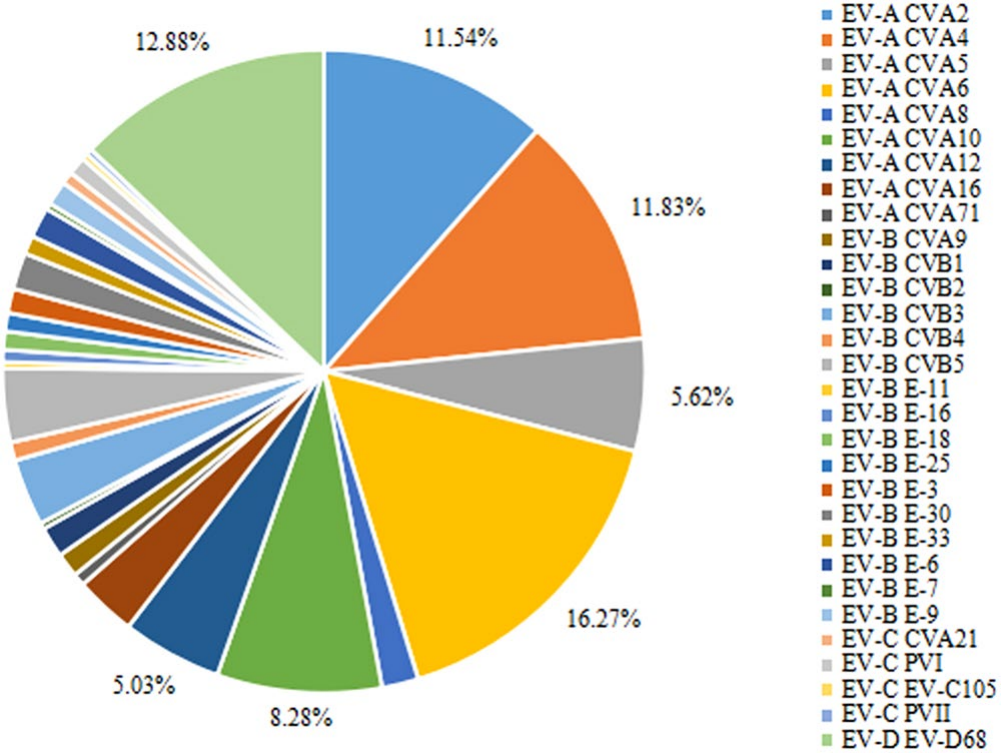
The serotypes of EV positive cases among EV-A to D.

A total of 21, 816 ARTI cases were collected from RVSS from January 2015 to December 2017. The median age of them was 36 years old (range: 1 day to 101 years). Among 21, 816 cases, 619 (2.84%) cases were enterovirus positive and 42 (0.19%) cases were EV-D68 positive. 359 cases were successfully amplified among 619 enterovirus positive cases, including 326 cases belonging to EV-A to D, 26 cases and 7 cases belonging to RV-A and RV-C respectively, and no case belonging to RV-B. Of 326 cases belonging to EV-A to D, they were genotyped to 30 serotypes including 9 of EV-A, 16 of EV-B, 4 of EV-C and 1 of EV-D and the dominant serotypes were CVA6, EV-D68, CVA4, CVA2, CVA10, CVA5 and CVA12, accounting for 16.27%, 12.88%, 11.83%, 11.54%, 8.28%, 5.62% and 5.03%, respectively (Fig. 1). No samples of EV-D68 co-infected with other enteroviruses were found.

The annual detection rates of EV-D68 were 0 (0/6, 644) in 2015, 0.53% (40/7, 522) in 2016 and 0.03% (2/7, 650) in 2017, respectively (Table 1). Twenty cases were aged younger than 18 years, 15 were aged 18 to 60 years and 7 were aged over 60 years. No statistically significant difference of EV-D68 positive rates was identified in the different age groups in 2016 (P = 0.062).

**Table 1:** Clinical samples screened for enterovirus and EV-D68 from January 2015 to December 2017 in Beijing, China.

Group	No. of clinical specimens N = 21816	No. of enterovirus positive (%) n = 619 (2.84)	No. of EV-D68 positive (%) n = 42 (0.19)
**Jan-Dec 2015**			
< 18 years old	2313	169 (7.31)	0
18 to 60 years old	2273	56 (2.46)	0
>60 years old	2058	21 (1.02)	0
**Jan-Dec 2016**			
<18 years old	2463	121 (4.91)	19 (0.77)
18 to 60 years old	2591	56 (2.16)	14 (0.54)
>60 years old	2468	37 (1.50)	7 (0.28)
**Jan-Dec 2017**			
<18 years old	2212	96 (4.34)	1 (0.05)
18 to 60 years old	2849	43 (1.51)	1 (0.04)
>60 years old	2589	20 (0.77)	0

### Temporal and geographic distribution of EV/EV-D68

**Figure 2:**
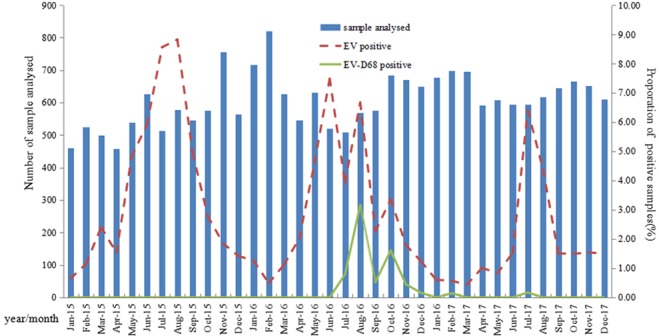
Proportions for EV and EV-D68 positive cases from January 2015 to December 2017.

Totally, 40 EV-D68 cases were detected during July-December in2016 and two peaks of EV-D68 infection occurred in August and October (Fig. 2). Eighteen of 38 (47.37%) EV positive cases were positive for EV-D68 in August, and 11 of 23 (47.8%) in October. They were distributed in 15 districts in Beijing, except Shunyi District. No epidemiological linkage was identified among the 40 patients (Supplemental Table 1).

### Clinical characteristics of patients infected EV-D68

The demographic and clinical characteristics of the 42 EV-D68 cases were shown in Tables 2, 3 and Supplemental Table 1. The median age of 42 EV-D68 cases was 23 years old (range: 0.3 to 86 years) and 28 (66.67%) cases were male.

**Table 2:** Clinical characteristics of the 42 cases of EV-D68 infection from January 2015 to December 2017 in Beijing, China.

Characteristic	No. (%) EV-D68		*P*	Total
	B3 (n = 27)	D3 (n = 15)		N = 42
**No**, **(%)**				
<18 years old	16 (59.26)	4 (26.67)	0.13	20 (47.62)
18 to 60 years old	7 (25.93)	8 (53.33)		15 (35.71)
>60 years old	4 (14.81)	3 (20.00)		7 (16.67)
Years median, (range)	7 (0.3–86)	35 (1–85)	/	23 (0.3–86)
**Sex**, **n (%)**				
M	16 (59.26)	12 (80.00)	0.31	28 (66.67)
F	11 (40.74)	3 (20.00)		14 (33.33)
**Epidemiology**, **n (%)**				
urban	4 (14.81)	5 (33.33)	0.51	9 (21.43)
suburb	10 (37.04)	6 (40.00)		16(38.10)
outskirts	13 (48.15)	4 (26.67)		17 (40.48)
**Diagnosis**, **n (%)**				
URTI	6 (22.22)	4 (26.67)	0.75	10 (23.81)
Pneumonia	21 (77.78)	11 (73.33)		32 (76.19)
**Clinical symptom**, **n (%)**				
Fever	19 (70.37)	11 (73.33)	0.84	30 (71.43)
Cough	25 (92.59)	15 (100.00)	0.53	40 (95.24)
Expectoration	19 (70.37)	9 (60.00)	0.52	28 (66.67)
Pharyngalgia	9 (33.33)	3 (20.00)	0.49	12 (28.57)
Dyspnea	4 (14.81)	5 (33.33)	0.24	9 (21.43)
Runny nose	6 (22.22)	1 (6.67)	0.39	7 (16.67)
rhinocleisis	4 (14.81)	2 (13.33)	1	6 (14.29)
Pectoralgia	0	1 (6.67)	0.36	1 (2.38)
Diarrhea	1 (3.70)	0	1	1 (2.38)
**Diagnosis**, **n (%)**				
Pulmonary consolidation	18 (66.67)	8 (53.33)	0.06	26 (61.90)
Interstitial lesion	4 (14.81)	0		4 (9.52)
Pleural effusion	0	2 (13.33)		2 (4.76)

**Table 3:** Nucleotide and amino acid sequence identity between subcalde D3 and other subtypes of EV-D68 strains. Based on comparisons of 9 complete genomes of subclade D3 strains and representative strains from other clades and subclades of EV-D68.

EV-D68 clade	Sequence identity range (%)			
	Nucleotide genome	Nucleotide, VP1	Amino acid, polyprotein	Amino acid, vp1
D3 vs. A	91.1–92.0	90.4–90.9	97.4–97.6	95.4–96.4
D3 vs. B1	89.0–89.4	87.3–88.5	96.5–96.9	94.1–95.1
D3 vs. B2	88.6–88.9	87.8–88.3	96.5–96.9	93.7–94.7
D3 vs. B3	90.1–90.4	91.3–94.4	96.4–96.7	93.4–94.3
D3 vs. C	90.1–90.7	88.3–89.3	96.6–97.0	93.5–94.5
D3 vs. D1	96.5–97.0	96.2–97.4	98.2–98.9	96.2–97.4
D3 vs. D2	90.5–92.4	93.6–94.3	97.4–97.6	93.6–94.3

There were 10 cases (23.81%) with URTI and 32 (76.19%) cases with pneumonia. Three cases, including one baby and two old men, were diagnosed with severe pneumonia and two of them were admitted to the ICU ward. No acute flaccid paralysis was found among 42 EV-D68 cases. They had been physically healthy without underlying diseases and immunocompromised before causing ARTI by EV-D68, and the prognosis was satisfactory after treatment

The common clinical characteristics included cough (95.24%), fever (71.43%), expectoration (66.67%) and pharyngalgia (28.57%). About 35.7% of EV-D68 infections had hyperpyrexia symptoms (39 °C–40.5 °C). The CXR findings showed 26 cases with the pulmonary consolidation, four cases with interstitial lesion and two cases with pleural effusion. Four cases who were diagnosed with pneumonia were accompanied with other pathogen infections (one with H3N2, one with respiratory syncytial virus and two with mycoplasma).

### Phylogenetic analysis of EV-D68 strains

A total of 42 VP1 gene region and 24 complete genome sequences of EV-D68 were obtained in Beijing from 2016 to 2017 and performed the phylogenetic analysis.

**Figure 3:**
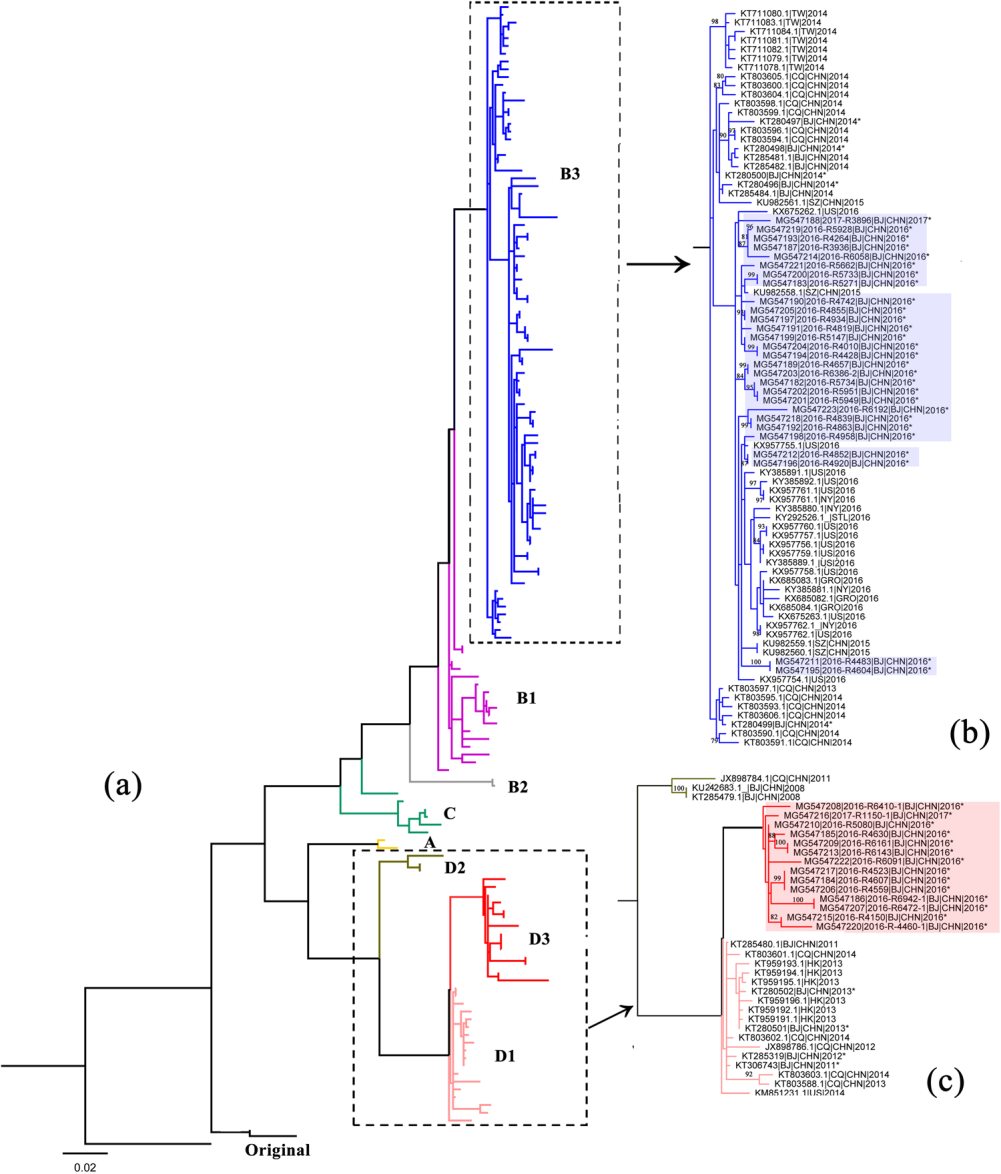
The phylogenetic tree of EV-D68 strains based on VP1 gene region from Beijing and other countries. Strains in this study were marked with shadow (GenBank Accession No. MG547182-MG547233). The phylogenetic relationships were estimated by the maximum-likelihood method with 1,000 the bootstrap replicates in MEGA6. GenBank Accession Numbers, the countries, the years and the clades were shown for each EV-D68 strains. Clade A was indicated in yellow, subclade B1 in purple, B2 in gray, B3 in blue, clade C in green, subclade D1 in pink, D2 in brown, and new subclade D3 in red. (**a**) The phylogenetic tree of EV-D68 strains; (**b**) the phylogenetic tree of strains in subclade B3; (**c**), the phylogenetic tree of strains in clade D.

The phylogenetic analysis of 146 representative VP1 gene region of EV-D68 showed 42 strains in this study fell into clades B and D. Twenty-six strains in 2016 and one strain in 2017 in this study belonged to subclade B3, and fourteen strains in 2016 and one strain in 2017 belonged to a new subclade D3 (Fig. 3 and Supplemental Fig. 1). Homology comparison showed the strains of subclade D3 VP1 gene region sharing 93.6–94.3% identity in nucleotide with subclade D2, and 96.2–97.4% with subclade D1 (Tab. 3).

**Figure 4:**
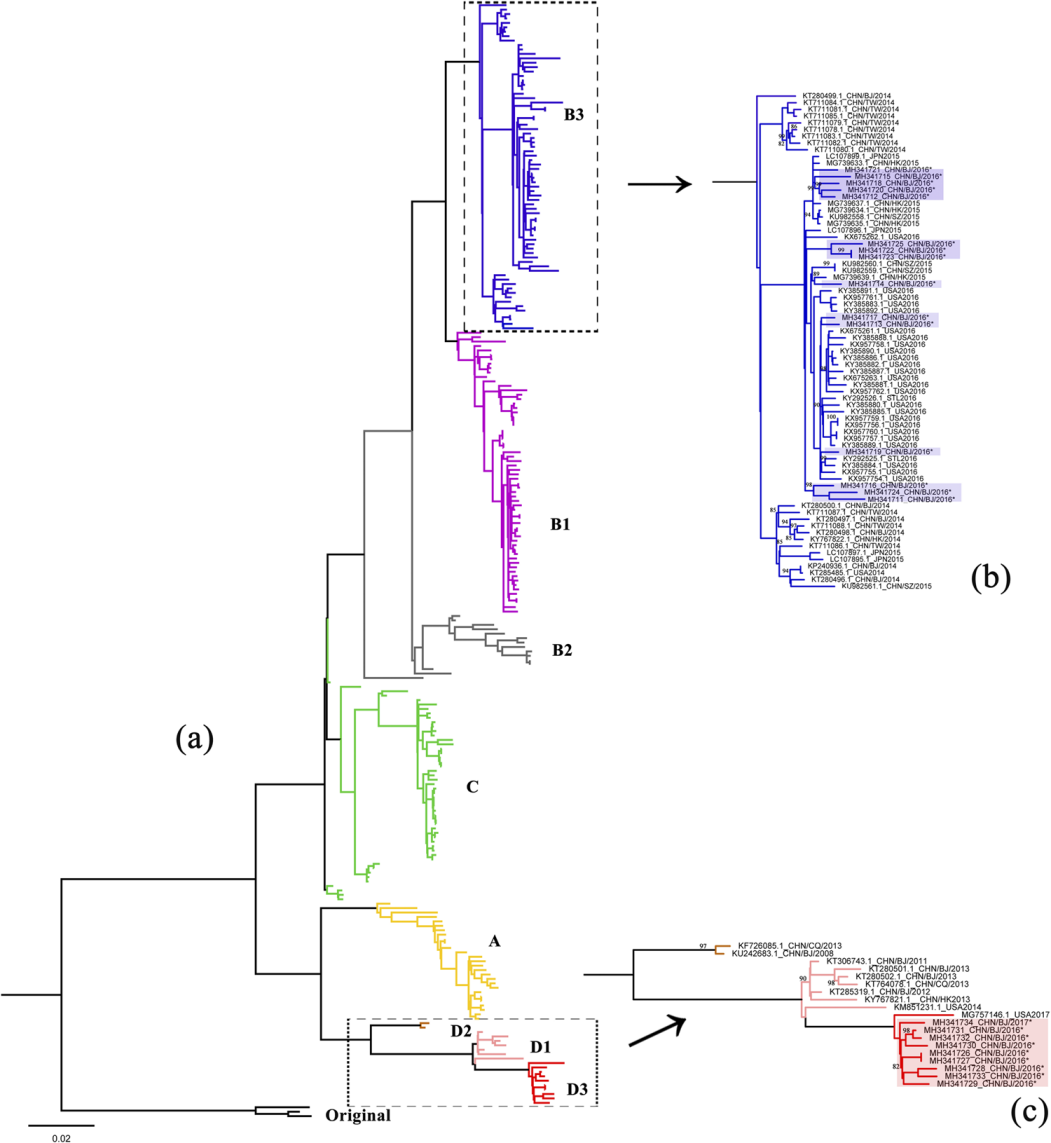
The phylogenetic tree of EV-D68 strains based on the complete genome sequences region from Beijing and other countries. The GenBank accession numbers of complete genomes in this study were from MH341711 to MH341734. The phylogenetic relationships were estimated by the maximum-likelihood method with 1,000 the bootstrap replicates in MEGA6. GenBank accession numbers, the countries, the years and the clades were shown for each EV-D68 strains. Clade A was indicated in yellow, subclade B1 in purple, B2 in gray, B3 in blue, clade C in green, subclade D1 in pink, D2 in brown, and new subclade D3 in red. (**a**) The phylogenetic tree of EV-D68 strains; (**b**), the phylogenetic tree of strains in subclade B3; (**c**), the phylogenetic tree of strains in clade D.

The phylogenetic analysis of 252 EV-D68 complete genome sequences available from GenBank, including representative strains of the outbreak in 2014 in USA, all strains in other years in USA and all strains in other countries, was shown in Fig. 4 and Supplemental Fig. 2. The subclade B3 from Beijing in China in 2016 were most close to recent EV-D68 strains detected in the United States of the outbreak in 2016, Japan in 2015 and Hong Kong, Shenzhen in China in 2015. Homology comparison showed 98.1–99.0% identity in nucleotide and 99.5–99.8% identity in amino acid between American strains and Chinese strains of subclade B3 in 2016. The subclade B3 also included 20 of EV-D68 additional strains in previously study from Beijing, Shenzhen and Taiwan in China between 2014 and 2015 and 2 from Japan in 2015, which were grouped into a separate cluster.

Similar to the tree based on VP1 gene region, the phylogenetic tree based on the complete genome that 9 of EV-D68 strains from this study and one strain detected in 2017 in USA (GenBank Accession No. MG757146) belonged to the new subclade D3, which were grouped into a new cluster separated from the subclades D1 and D2 strains of clade D over the world before 2017 with 90.5–97.0% identity in nucleotide. Homology comparison showed fifteen strains of subclade D3 in this study sharing 98.9–99.9% identity in nucleotide and 99.5–99.7% identity in amino acid with each other, and 98.2–98.5% identity in nucleotide and 99.1%–99.2% identity in amino acid with the American strain (GenBank Accession No. MG757146).

**Figure 5:**
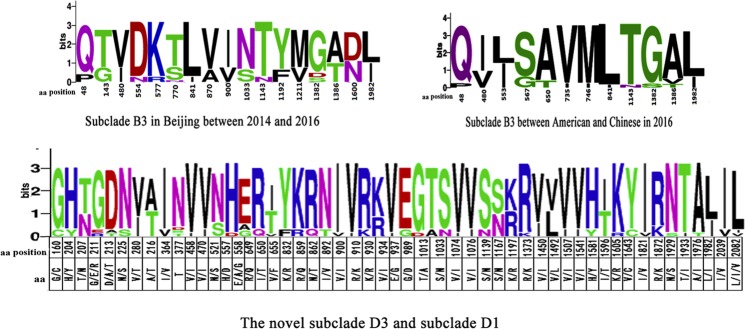
Amino acid (aa) polymorphisms of EV-D68 subclade B3 and D3 based on the entire polypeptide sequences of 2190 aa. The graphical representation was generated using Weblogo3. The height of symbol indicates the relative frequency of the corresponding Amino acid. Amino acid position was based on the EV-D68 prototype strain Fermon (GenBank Accession No. AY426531).

On basis of the complete polypeptide sequences of 2190 amino acids, a total of 52 amino acid polymorphisms were identified between subclade D1 and D3, which may further support the new cluster of D3. There were 17 amino acid polymorphisms identified from subclade B3 in Beijing between 2014 and 2016 and 12 amino acid polymorphisms identified from subclade B3 between American and Chinese strains in 2016 (Fig. 5).

### Molecular epidemiology of EV-D68 circulating in Beijing

The analysis of our sequences in this study and our prophase study^23^ collected from RVSS showed subclades B1, B3, D1 and D3 appeared to dominate in different years in Beijing between 2011 and 2017. Both subclade D1 and B1 were co-circulating in 2011, only subclade D1 was sequentially detected in 2012 and co-circulated with subclade B1 in 2013 once again, but not detected in the subsequent years. However, subclade B3 was detected for the first time as single prevalent lineage in 2014. The new subclade D3 was first detected in 2016 and co-circulated with subclade B3 during 2016 to 2017.

Between 2011 and 2014, EV-D68 infections appeared mainly in the people younger than 18 years of age (11/12) in our previously study^18^. However, more of EV-D68 infections appeared in the adult group from 18 to 60 years of age (15/42) and elderly group over 60 years of age (7/42) in Beijing, that 11 cases were caused by subclade B3 and 11 by subclade D3. No statistical difference of age was identified among EV-D68 positive patients caused by subclades B3 or D3 (P = 0.130).

Of 27 cases caused by subclade B3, 6 cases were diagnosed with URTI and 21 cases with pneumonia, including 1 cases with severe pneumonia. Of 15 cases caused by the new subclade D3 in 2016, 4 cases were diagnosed with URTI and 11 cases with pneumonia, including 2 cases with severe pneumonia. There was no statistical differences of the proportions on pneumonia or severe pneumonia caused by subclade B3 or D3 (both P > 0.05) (Table 2).

## Discussion

The outbreaks of ARTI caused by EV-D68 had been reported in various countries in 2014, such as the United States, Canada, Brazil, Norway, Netherlands and other countries^19^. Recent study in USA showed a significant decrease of EV-D68 detection in 2015, and subsequently another outbreak occurred in 2016^24^. The study in the Netherlands showed very limited activity of EV-D68 was observed in 2015 and an upsurge in 2016^25^. The outbreak of EV-D68 in Stockholm Sweden with 33% EV-D68 positive samples during the peak week was reported in 2016^27^.

To detect ARTI caused by EV-D68, BJCDC has established RVSS for timely monitoring and retrospective analysis since 2014. The general trend of EV-D68 epidemic in China was similar to that of other countries in the world. From 2011 to 2014, 12 EV-D68 positive cases were confirmed sporadically by BJCDC in Beijing, of which 5 cases was detected in 2014^21,22^. Nineteen cases of EV-D68 positive were detected in Chongqing between 2012 and 2014, including 13 cases in 2014^20^. Fifty-seven cases of EV-D68 positive were detected in Taiwan between 2007 and 2014, including 8 cases in 2014^34^. Rarely EV-D68 positive cases had been reported in China in 2015 and only 4 cases were confirmed in Shenzhen^35^. In this study, the upsurge of EV-D68 with 40 cases confirmed was in 2016, and significant decreased in 2017 with only 2 cases confirmed. In recent years, there was a trend that the peak of EV-D68 appeared every two years.

However, the circulation of diverse EV-D68 subclades in China were slightly different from that in the world. Notable, it showed a new subclade of EV-D68 strains in Beijing from 2016 to 2017. One study by Cyril *et al*. showed that 2.6–7.1% diversity in nucleotide identity of EV-D68 VP1 gene region could be divided into different subclades^31^. The diversities of EV-D68 VP1 gene region in nucleotide were 2.6–3.8% between subclade D1 and D3, and 5.7–6.4% between subclade D2 and D3. Therefore, we defined the new subclade of EV-D68, subclade D3. In this study, the phylogenetic analysis showed subclade B3 strains with highly homologous were grouped into the same evolutionary branch with strains in other countries in 2016, but in separated evolutionary branches with the strains in Beijing, Hong Kong, Shenzhen and Taiwan during 2014 to 2015.

In the United States, two subclades, a major subclade B1 and a minor subclade B2, had co-circulated during the outbreak in 2014^14^ and subclade B3 had emerged and caused outbreak in 2016^24^. In the Netherlands, both a major subclade B1 and a minor subclade D were detected in 2014, and only subclade B3 upsurged in 2016^25^. In addition, the subclade B3 was detected in Sweden in 2016^27^. The phenomenon of different subclades of EV-D68 circulating in China was illustrated as follows. In Hong Kong, EV-D68 subclades B1, D1 and D2 showed a state of sporadic from 2010 to 2013 and a new subclade B3 was circulating in 2014^28,31^. In Beijing, EV-D68 subclades B1 and D1 showed a state of sporadic from 2011 to 2013 and a new subclade B3 circulating in 2014 caused the increase of EV-D68 infection. An upsurge of EV-D68 new subclade D3 and subclade B3 in Beijing in 2016. Whether the emergence of the new subclade D3 of EV-D68 in Beijing would cause an outbreak should be issues worthy of high concern.

Homology analysis showed multiple mutations in the 2C protein region which inhibits NF-κB mediating the adaptive immunity and innate immunity of the body to enterovirus^36^. Therefore, the phenomenon of the high mutation in 2C protein region needed to be further study.

Several clinical characteristics should be noticed in our patients who had EV-D68 subclades B3 and D3 infections in this study with the comparison of previously studies. First of all, there were the major URTI and a minor pneumonia causing by EV-D68 in China^20–22,34–36^. But 32 cases in this study were diagnosed with pneumonia including 3 cases with severe pneumonia. In addition, studies showed the majority of EV-D68 positive cases were children, elderly patients and a few adult around world^12,19,21,28^. In Hong Kong, their study firstly demonstrated EV-D68 could caused severe respiratory diseases in elderly people^28^. Several reports from China, US, Sweden, Netherlands, Denmark also identified a few adults or elderly people with ARTI, but there was no statistical analysis to show the characteristics of EV-D68 distribution among different age groups of people^21,24–28^. Our demonstrated EV-D68 could cause ARTI among all age groups of people equally. Last, Subclades B3 and D3 could lead lobes to pulmonary consolidation and interstitial lesion, even pleural effusion.

In conclusion, our study revealed an upsurge of EV-D68 in Beijing in 2016, and the new subclade D3 emerged in 2016 co-circulated with subclade B3 during 2016 to 2017 period and the patients of EV-D68 infection among all age groups. The prevalence and genetic variations of EV-D68 in China was of great significance to EV-D68 prevention in the world. Whether the emergence of new subclade D3 would cause outbreaks in the future should arouse our attention.

## Methods

### Patients

RVSS established by the Beijing Center for Disease Prevention and Control (BJCDC) in 2011 and now covers 30 sentinel hospitals distributing in all 16 districts of Beijing. This surveillance has obtained ethics approval from the Ethics Committee at BJCDC and experiments were performed in accordance with relevant guidelines and regulations. At recruitment, written informed consent had been obtained from the patient or the guardian. For research involving human participants under the age of 18 years (including donors of tissue samples), informed consent had been obtained from a parent and/or legal guardian. All clinical specimens were collected from RVSS. Clinical specimens, including nasopharyngeal swabs and sputum, were collected from acute respiratory tract infection (ARTI) and sent to BJCDC for laboratory diagnostic testing. ARTI cases in this study included upper respiratory tract infection (URTI) and pneumonia (including severe pneumonia). Diagnosis according to the criterions established by Pediatrics Society and Respiratory Society, Chinese Medical Association^37–39^.

### Real-time PCR for detection of EV-D68 and other enterovirus

Total nucleic acid (RNA and DNA) was extracted from the clinical specimens by Thermo Scientific™ KingFisher™ Flex Magnetic Particle Processors (Thermo Fisher). EV-D68 and other enteroviruses were simultaneously detected in one tube with a commercial real-time RT-PCR kit (cat. no. CN08-4G-100, Jiangsu uninovo biological technology company, China). A panel of respiratory pathogens, including influenza virus A (pandemic influenza virus H1N1, seasonal influenza A virus H3N2) and B, respiratory syncytial virus, parainfluenza virus 1 to 4, human adenovirus, human rhinovirus, human metapneumovirus, human coronavirus (NL63, OC43, 229E and HKU1), human bocavirus, mycoplasma and chlamydia, were also detected in these specimens with commercial real-time RT-PCR kits (cat. no. CN12-33-100 and cat. no. CN09-4-100, Jiangsu Uninovo Biological Technology Co. Ltd., China). The serotypes of enteroviruses were identified with the the seminested and conventional RT-PCR method for amplification of VP1 sequences (cat. no. C81401180, Invitrogen GoldScript, United States)^40^.

### Phylogenetic analysis of VP1 gene regions and complete genomes

The fragments of EV-D68 positive specimens were amplified and sequenced with EV-D68 specific primers^23^. The consensus sequences were assembled using BioEdit software, version 7.0.9. The phylogenetic tree of VP1 gene region was estimated using the GTR + G model in maximum-likelihood method and the complete genome was estimated using GTR + G + I model in maximum-likelihood method, with bootstrap analysis of 1,000 replicates, in MEGA software, version 6.0. The amino acid polymorphisms and substitutions identified by sequence analysis were plotted on graphs using the Weblogo, version 3.0.

### Statistical analysis

The differences of EV-D68 positive cases among different age groups were performed using chi-square test. The comparison of EV-D68 different subclades proportions in different age groups were performed using fisher’s exact test. The comparison of respiratory diseases by different subclades of EV-D68 were performed using fisher’s exact test. A two-sided P value of less than 0.05 was considered to be statistically significant. All analyses were performed using SPSS software, version 17.0.

## Supplementary information

The phylogenetic tree of VP1 gene regions and complete genomes of EV-D68 strains

Clinical characteristics of the 42 cases of EV-D68 infection from January 2015 to December 2017 in Beijing
