# Supplementary material for: Upsurge of Enterovirus D68 and Circulation of the New Subclade D3 and Subclade B3 in Beijing, China, 2016
Source: Sci Rep. 2019 Apr 15;9:6073. doi: 10.1038/s41598-019-42651-7 (PMC6465342; doi:10.1038/s41598-019-42651-7)
Supplement: Supplementary file 1 — The phylogenetic tree of VP1 gene regions and complete genomes of EV-D68 strains [file 41598_2019_42651_MOESM1_ESM.pdf]

Title page

Upsurge of Enterovirus D68 and Circulation of the New Subclade D3 and Subclade B3 in Beijing, China, 2016

Lingyu Shen[1,2], Cheng Gong[2], Zichun Xiang[3], Tiegang Zhang[2], Maozhong Li[2], Aihua Li[2], Ming Luo[1], Fang Huang[1,2*]

[1]College of public health, Capital Medical University, Beijing, China. No.10 West, You'anmen Avenue of Fengtai district, Beijing 100069, P. R.China.

[2]Beijing Center for Disease Prevention and Control. No.16, Hepingli Middle Avenue of Dongcheng district, Beijing 100013, P. R. China.

[3]MOH Key Laboratory of Systems Biology of Pathogens and Christophe Mérieux Laboratory, IPB, CAMS-Foundation Mérieux, Institute of Pathogen Biology (IPB), Chinese Academy of Medical Sciences (CAMS) & Peking Union Medical College, P. R.China.

Correspondence and requests for material should be addressed to F.H. (email: hhffxdd@126.com)

Tel: 86-10-64407032;

Fax: 86-10-64407032;

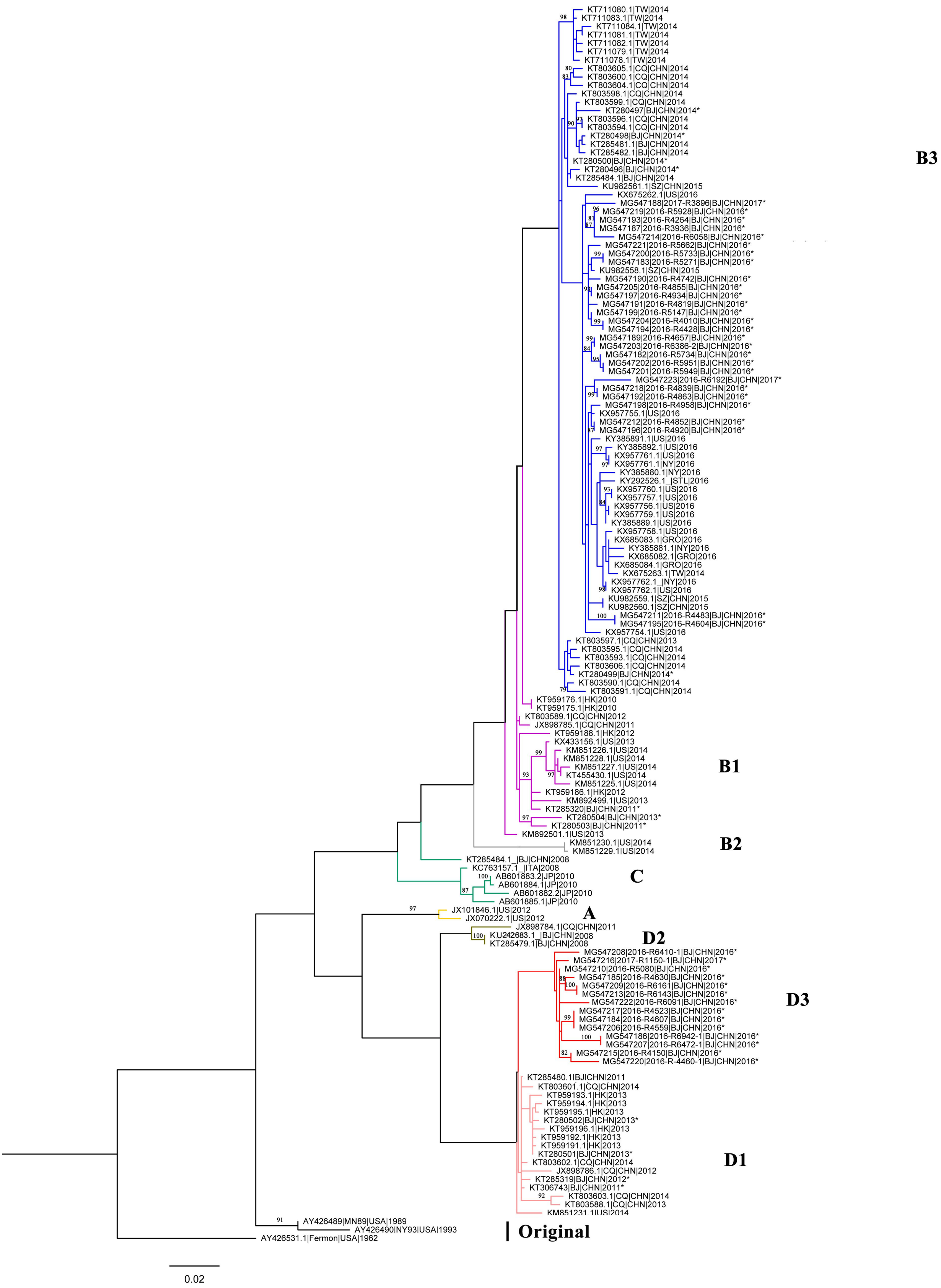

Supplemental Fig.1 The phylogenetic tree of VP1 gene regions of EV-D68 strains

GenBank accessions of strains in this study, No. MG547182-MG547233. The phylogenetic relationships were estimated by the maximum-likelihood method with 1,000 the bootstrap replicates in MEGA6. GenBank accession numbers, the countries, the years and the clades were shown for each EV-D68 strains. Clade A was indicated in yellow, subclade B1 in purple, B2 in gray, B3 in blue, clade C in green, subclade D1 in pink, D2 in brown, and new subclade D3 in red.

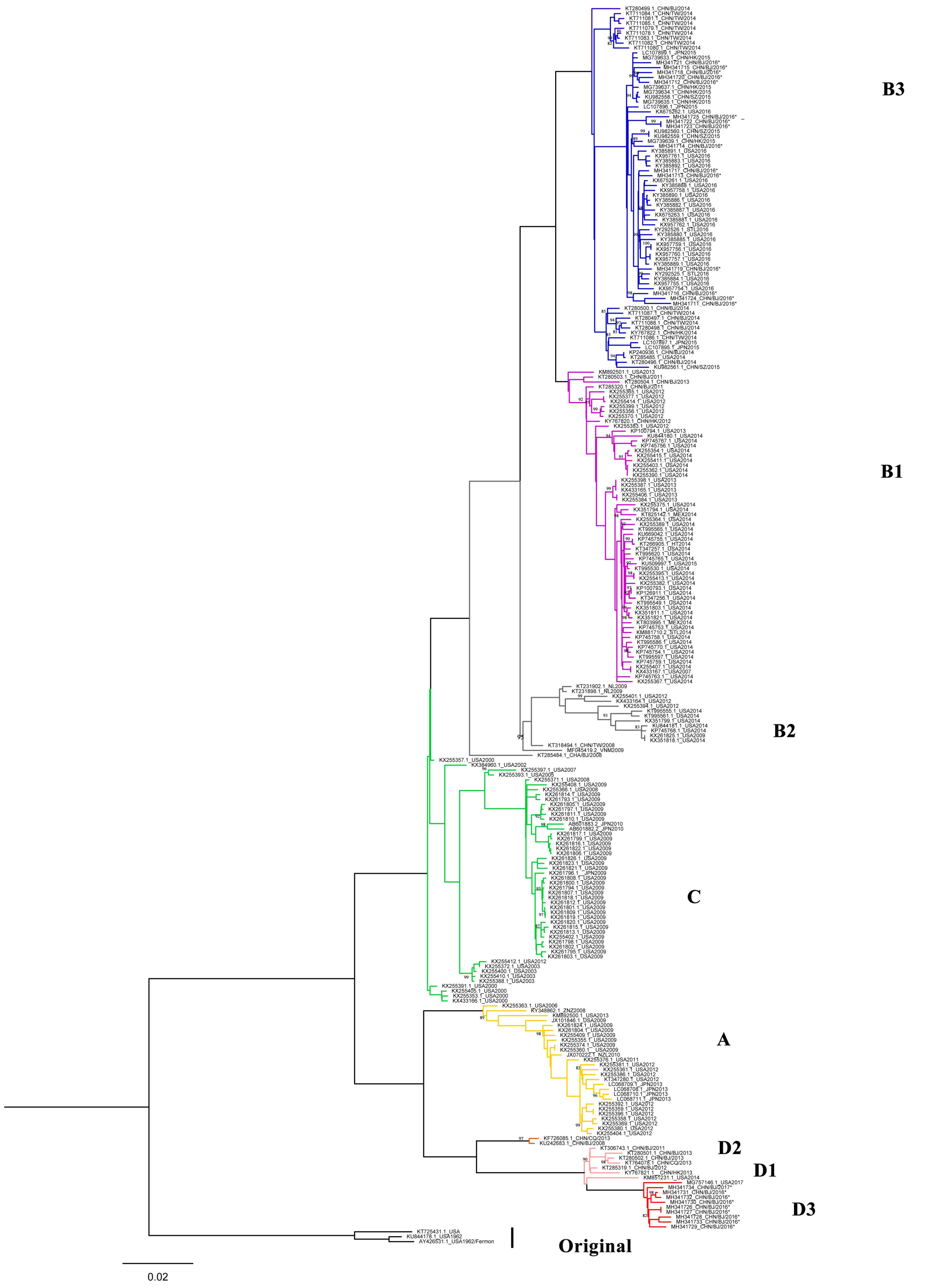

Supplemental Fig.2 The phylogenetic tree of complete genomes of EV-D68

GenBank accessions of strains in this study, No. MH341711 to MH341734. The phylogenetic relationships were estimated by the maximum-likelihood method with 1,000 the bootstrap replicates in MEGA6. GenBank accession numbers, the countries, the years and the clades were shown for each EV-D68 strains. Clade A was indicated in yellow, subclade B1 in purple, B2 in gray, B3 in blue, clade C in green, subclade D1 in pink, D2 in brown, and new subclade D3 in red.
